# Rituximab therapy in pemphigus and other autoantibody-mediated diseases

**DOI:** 10.12688/f1000research.9476.1

**Published:** 2017-01-27

**Authors:** Nina A. Ran, Aimee S. Payne

**Affiliations:** 1 Department of Dermatology, University of Pennsylvania, 1009 Biomedical Research Building, 421 Curie Boulevard, PA, USA

**Keywords:** Pemphigus, desmoglein, rituximab, autoantibody-mediated diseases

## Abstract

Rituximab, a monoclonal antibody targeting the B cell marker CD20, was initially approved in 1997 by the United States Food and Drug Administration (FDA) for the treatment of non-Hodgkin lymphoma. Since that time, rituximab has been FDA-approved for rheumatoid arthritis and vasculitides, such as granulomatosis with polyangiitis and microscopic polyangiitis. Additionally, rituximab has been used off-label in the treatment of numerous other autoimmune diseases, with notable success in pemphigus, an autoantibody-mediated skin blistering disease. The efficacy of rituximab therapy in pemphigus has spurred interest in its potential to treat other autoantibody-mediated diseases. This review summarizes the efficacy of rituximab in pemphigus and examines its off-label use in other select autoantibody-mediated diseases.

## Introduction

Autoimmunity occurs when the body’s immune system mistakenly attacks self rather than foreign pathogens, leading to end-organ damage. B cells play a role in autoimmunity through several potential mechanisms, including antigen presentation, regulation of inflammation, and production of autoantibodies. These autoantibodies directly cause disease in several disorders, including pemphigus.

The critical role of B cells in autoimmune disorders has prompted interest in the use of B-cell-depleting therapies such as rituximab, a chimeric monoclonal antibody against the B cell surface antigen CD20. Of note, rituximab has striking efficacy in pemphigus, a finding mostly attributed to the central role of autoreactive B cells and autoantibodies in this disease. Rituximab has also been shown to downregulate autoreactive T helper cells in pemphigus patients- either by reducing B-cell-mediated antigen presentation or through direct depletion of CD20 ^+^ T cells; the latter has been identified at similar rare frequencies in patients with multiple sclerosis, rheumatoid arthritis (RA), and healthy individuals, although their pathophysiologic significance to disease onset and remission after rituximab remains unclear ^1– 3^. Nevertheless, the success of rituximab in pemphigus invites a reappraisal of its therapeutic efficacy in other autoantibody-mediated diseases. This review uses pemphigus as a paradigm to discuss the pathophysiology of select autoantibody-mediated diseases and to evaluate the role of rituximab in their treatment.

## Pemphigus: a paradigm for autoantibody-mediated diseases

In pemphigus, autoantibodies to desmoglein (Dsg) skin cell adhesion proteins cause potentially severe epithelial blistering ^4, 5^, which can lead to death from malnutrition, dehydration, and infection. There are two major subtypes of pemphigus: pemphigus vulgaris (PV), which is characterized by autoantibodies to Dsg3, and pemphigus foliaceus (PF), characterized by autoantibodies to Dsg1.

Laboratory studies and clinical observations have established that the anti-Dsg antibodies in pemphigus sera are by themselves the disease-causing agents. Dsg1 and Dsg3 ELISAs have high sensitivity and specificity (98–100%) for disease, indicating that pemphigus does not occur in the absence of anti-Dsg antibodies and, furthermore, disease activity correlates with serum autoantibody titer ^6– 8^. Direct evidence for the pathogenicity of anti-Dsg antibodies comes from early observations that transplacental transfer of autoantibodies from mothers with PV leads to neonatal pemphigus ^9– 12^. Definitive evidence derives from experiments showing that affinity purified anti-Dsg antibodies as well as recombinant monoclonal anti-Dsg antibodies cause characteristic pemphigus skin blisters ^13– 18^ and conversely that removal of anti-Dsg antibodies from pemphigus serum abolishes its pathogenicity ^19, 20^. Moreover, anti-Dsg autoantibodies cause blister formation in human skin and animal models even as monovalent antibody fragments ^15, 17, 21, 22^, indicating that antigen cross-linking or Fc-mediated functions are not required for pathogenicity, even though such mechanisms can contribute to blister formation ^23^. Autoimmunity against other antigens in pemphigus has also been described ^24– 28^. Regardless, these data collectively and definitively establish pemphigus as an autoantibody-mediated, not just an autoantibody-associated, disease. This conclusion spurs the rationale for evaluating the efficacy of B-cell-depleting agents in pemphigus.

### Efficacy of rituximab in pemphigus

Rituximab has emerged as an effective therapeutic option for pemphigus patients. Nearly all patients (95–100%) experience initial disease control ^29, 30^. A recent meta-analysis of 578 cases showed a complete remission (CR) rate of 76% after rituximab ^31^, which included patients remaining on systemic immunosuppressive therapies; CR rates of 59% and 100% were reported in prospective studies, with the former using a more stringent definition of CR ^30, 32^. The rate of relapse generally increases with length of clinical follow-up, ranging from 40–81%, with long-term rates of CR off therapy observed in 39–45% of patients ^31, 32^. One prospective study of rituximab and intravenous immunoglobulin (IVIg) in 11 patients reported 100% of patients achieving CR off therapy after long-term follow up ^33^.

The optimal dosing for rituximab in pemphigus has not been established. Because rituximab was initially approved for lymphoma, early pemphigus treatment protocols more often used the lymphoma dosing regimen (375 mg/m ^2^ weekly for four weeks). However, because the B cell burden in autoimmune disease is much lower than that in lymphoma, several studies have evaluated the RA regimen (two 1,000 mg doses given two weeks apart). A meta-analysis found no significant difference in CR rates between the two treatment regimens, although higher-dose protocols were associated with a significantly longer duration of disease remission ^31^. Additionally, relapse after rituximab has been shown to be associated with the same anti-Dsg B cells observed during active disease ^34^, indicating that relapse is due to incomplete B cell depletion; thus, higher-dose regimens that are more likely to achieve complete B cell depletion should offer the highest chance for long-term CR off therapy.

Several findings suggest the use of rituximab as a component of first-line therapy for disease. These include an association between disease duration prior to rituximab and rate of CR ^35^, as well as a 100% rate of long-term CR off therapy in five patients who received rituximab as first-line therapy ^32^. A randomized, open-label trial comparing rituximab and moderate-dose corticosteroids to high-dose corticosteroids as first-line therapy has recently completed, with study results pending publication (ClinicalTrials.gov ID: NCT00784589).

## Other autoantibody-mediated diseases and the pemphigus paradigm

Several other autoantibody-mediated diseases occur in humans. However, they may vary from the pemphigus paradigm in the diversity of autoantigens, the sensitivity and specificity of the autoantibodies, and the downstream effects of antibody binding. Nevertheless, an increasing number of studies have shown similarly promising results for the use of rituximab in many of these diseases, in particular neuromyelitis optica (NMO), thrombotic thrombocytopenic purpura (TTP), and myasthenia gravis (MG). The published literature includes a few hundred patients for each of these three diseases and has found notable improvements in remission and relapse rates. While randomized controlled trials are needed to confirm these initial findings, these three diseases represent areas of particular potential for B cell depletion strategies.

### Neuromyelitis optica

NMO is characterized by inflammation, demyelination, and axonal injury to the spinal cord and optic nerve. The discovery that patients have antibodies against the astrocyte water channel aquaporin-4 (AQP4) helped distinguish NMO patients from those with multiple sclerosis ^36– 38^, and these antibodies are now a central part of NMO diagnosis ^39^. While the antibodies are nearly 100% specific, their sensitivity varies by assay and study (48–87%) ^40, 41^. Data have not consistently shown a correlation between antibody levels and disease activity or risk of relapse ^42, 43^. However, the findings of select studies suggest that anti-AQP4 antibodies may have prognostic value: antibody titers correlate with extent of transverse myelitis, antibody levels increase sharply before relapse in some patients, and positive titers during the initial episode of transverse myelitis may predict recurrent attacks ^44– 46^.

Strong support for the pathogenicity of anti-AQP4 antibodies come from studies using monoclonal recombinant antibodies, purified antibodies, or patient serum ^47– 50^. Passive transfer of antibodies into mouse and rat experimental autoimmune encephalomyelitis (EAE) models exacerbates EAE symptoms and causes characteristic NMO lesions, including demyelination and/or loss of astrocytes with a decrease in astrocyte surface AQP4 expression in areas adjacent to astrocyte depletion. Removal of anti-AQP4 antibodies from patient sera markedly reduced the pathology ^49^. In addition, plasma exchange has been used with success in steroid-refractory NMO patients ^51– 53^ and as first-line therapy ^54^. Still, several aspects of NMO pathogenesis require clarification, including whether anti-AQP4 antibodies are present in the central nervous system at the onset of disease. In addition, the observation that anti-AQP4 antibodies have produced pathology only in EAE models with prior T-cell-mediated inflammation has sparked interest in how the cellular immune response mediates disease ^47, 49^. A recent study found that transfer of anti-AQP4 reactive T cells into mice led to inflammatory infiltrate and demyelination, without the loss of astrocytes and AQP4 observed with antibody transfer ^55^; thus, the authors postulate that T cells may initiate and localize the autoimmune response as well as regulate downstream immune mediators including antibodies. Collectively, these data indicate that NMO antibodies are necessary for specific aspects of disease pathology, but the data supporting their sufficiency for disease induction have not been as clearly established.

Rituximab is commonly used to prevent relapse in NMO. Following a seminal study in eight patients ^56^, promising results have led to larger studies that have included more AQP4 IgG seropositive patients using a regimen of rituximab induction (either lymphoma or RA protocol) followed by maintenance infusions. The optimal rituximab maintenance strategy has not been established. Some studies used a fixed schedule (e.g. re-treatment every 6–9 months) ^57, 58^ and chose to re-treat sooner if CD19 ^+^ B cell count rose ^59^, while others re-treated based solely on memory B cell repletion ^60^. Studies with patients previously on other therapies found a 50–70% rate of relapse-free disease and a decrease in annualized relapse rate (ARR) of up to 96% ^57, 59– 61^. Rituximab has also been effective as initial therapy (84% rate of relapse-free disease; 97% reduction in ARR) ^58^, and the European Federation of Neurological Societies has recommended rituximab as first-line treatment for NMO ^62^.

### Thrombotic thrombocytopenic purpura

TTP is a life-threatening thrombotic microangiopathy that arises when the metalloprotease ADAMTS13 does not cleave von Willebrand factor (vWF); this leads to the persistence of ultra-large vWF multimers that promote platelet aggregation and vessel occlusion. Acquired idiopathic TTP stems from autoantibodies against ADAMTS13. Inhibitory antibodies can prevent proteolysis by binding to the domain of ADAMTS13 that interacts with vWF ^63, 64^. More rarely, non-inhibitory antibodies that have no *in vitro* neutralization effect can still interfere with ADAMTS13 activity *in vivo* by promoting its clearance or preventing its binding to factors such as endothelial cells ^65^. Anti-ADAMTS13 antibodies have been detected in >95% of patients with severely deficient ADAMTS13 levels (i.e. <10% normal) ^66, 67^. However, the antibodies are less specific, as they have also been found in patients with systemic lupus erythematosus and anti-phospholipid antibody syndrome, as well as in healthy individuals ^66, 68^. Conflicting data exist regarding an association between antibody titer and disease course ^69– 71^.

Antibody pathogenicity was demonstrated by mouse monoclonal antibodies against ADAMTS13 that triggered TTP in baboons ^72^. New data also show that expression of inhibitory human single chain variable fragment (scFv) antibodies in mice results in features of TTP, further suggesting that antibody effect does not necessarily require Fc-mediated mechanisms ^73^. Additional support comes from the successful use of plasmapheresis to remove inhibitors and replace functional ADAMTS13, which is associated with an 80–90% survival rate and is used as standard first-line therapy ^74– 76^.

Rituximab has been used in roughly 250 TTP patients in the literature, either in refractory patients, as initial treatment, or during remission to prevent relapse ^77^. In a prospective study of 22 TTP patients with refractory disease, rituximab led to faster achievement of remission and higher rates of remission at 35 days (100%) compared to historic controls (78%) ^78^. While rituximab led to lower relapse rates at one year (0%) compared to controls (9%), the long-term relapse rate did not differ between the groups. When used in the initial treatment of acute TTP, rituximab led to lower relapse rates at one year compared to historic controls (0% vs. 16%), as well as during follow-up (11% vs. 55%), although the follow-up duration was longer in the control group ^79^. Lastly, studies have used rituximab maintenance dosing during remission to prevent relapse in patients with severe ADAMTS13 deficiency. In a recent cross-sectional study, those on rituximab had lower rates of relapse during the follow-up period (10%) compared to historic controls (39%), although follow-up for the controls was again longer. In general, rituximab is associated with an increase in ADAMTS13 activity and a decrease in inhibitor levels. Currently, rituximab is recommended for use in patients refractory to plasmapheresis and steroids and as initial treatment in severe forms of acute TTP ^80^.

### Myasthenia gravis

MG was the first autoantibody-mediated neurologic disease to be discovered ^81^, and the disease has two main autoantigenic targets. Roughly 80–90% of patients have antibodies against the nicotinic acetylcholine receptor (AChR); these cause complement-mediated destruction ^82– 85^, crosslinking-induced activation and downregulation ^86^, or direct interference with ACh binding of the AChR ^87^, resulting in muscle fatigue and weakness. While autoantibody titers are not predictive of disease course ^88^, the causal role of autoantibodies has long been established: transplacental transfer of antibodies from mothers with myasthenia to the neonate can cause transient muscle weakness, and passive transfer of patient serum to mice leads to smaller miniature endplate potentials (MEPPs) and reduced AChR density ^81, 89^.

MG can also be caused by antibodies against muscle-specific receptor tyrosine kinase (MuSK), a transmembrane protein found on the post-synaptic membrane. Anti-MuSK antibodies are found in 40–70% of myasthenia patients lacking anti-AChR antibodies, although a lower prevalence has been observed in a few studies, particularly those in Asian ethnic groups ^90– 92^. As the antibodies are mostly IgG4 and do not fix complement, immune complexes are not found in the synapse ^93, 94^. Growing evidence has supported, though not firmly established, their pathogenic role. While muscle biopsies from MuSK-Ab-positive patients had smaller MEPPs, they did not show the reduction in AChR density or the striking synaptic structural changes observed in AChR-Ab-positive patients ^94, 95^. However, mice injected with purified anti-MuSK antibodies exhibit changes in synapse morphology (including reduced AChR density) and muscle weakness ^96^. In addition, the high success rate of plasma exchange in MuSK-Ab-positive patients supports a pathogenic role of the antibody ^97^.

Rituximab has been used in MG patients refractory to conventional immunosuppressive therapy. Roughly 200 MG patients have received rituximab in the literature, which predominantly consists of case reports and case series. Compared to AChR-Ab-positive patients, those with anti-MuSK antibodies have a higher response rate. as well as more marked and sustained improvement ^98, 99^, leading investigators to propose that rituximab may have benefit earlier in the treatment of these patients ^99^. A recent meta-analysis showed an overall response rate of 84% and found non-significant differences in the response rates among anti-MuSK-positive patients (89%), anti-AChR-positive patients (80%), and double seronegative patients (86%) ^100^. The first double-blind randomized controlled trial of rituximab in anti-AChR-positive MG patients is ongoing (ClinicalTrials.gov ID: NCT02110706), and a second trial in patients with new onset, generalized MG is currently recruiting (ClinicalTrials.gov ID: NCT02950155).

### Graves’ disease

Thyrotropin receptor autoantibodies (TRAb), or thyroid stimulating hormone receptor (TSHR) autoantibodies, play a critical role in autoimmune thyroid disease and are classified as stimulating, blocking, or neutral (although the latter has been shown to modulate downstream TSHR signaling). The hyperthyroidism characteristic of Graves’ disease is caused by stimulatory TRAb, which are observed in nearly all patients ^101^. While antibody levels decline with therapy, and while antibody persistence has been linked to a higher risk of relapse, TRAb level is not considered a reliable predictor of treatment response ^102^. The pathogenicity of TRAb has long been established by passive transfer experiments with patient serum, and by the ability of maternal autoantibodies to cause transient hyperthyroidism in the neonate ^103, 104^. Additional support comes from the recent isolation of two stimulatory human monoclonal TRAbs – M22 and K1-18. These have similar TSHR binding affinity as antibodies from patient sera, with thousands-fold greater potency. The Fab fragment of M22, but not of K1-18, has similar characteristics to the intact autoantibody ^105– 107^.

Case series and open-label studies of rituximab in Graves’ orbitopathy (GO) have shown a >90% rate of disease inactivation ^108^. Two double-blind randomized controlled trials have been published to date, both in euthyroid patients with active, moderate-to-severe GO. The first study in 25 patients found no difference in clinical improvement between rituximab and placebo ^109^, while the second trial in 31 patients found greater clinical improvements with rituximab versus methylprednisolone ^110^. The authors of the second trial note that the discrepancy could be due to differences in the study populations, including number of patients, duration of disease, and prior steroid use. Still, in light of this inconsistency as well as previous data, rituximab is recommended as a second-line option for patients who are unresponsive to corticosteroids ^111^.

### Immune thrombocytopenic purpura

Immune thrombocytopenic purpura (ITP) results from autoantibodies against multiple platelet surface proteins, including glycoproteins (GP)1b/IX and GPIIa/IIIb, leading to platelet destruction ^112, 113^. Unlike pemphigus, anti-platelet antibodies have low sensitivity for the disease, with detection in roughly half of patients ^114– 116^. *In vitro* studies have shown that these antibodies bind complement, inhibit megakaryocytopoiesis, and hinder proplatelet formation ^117, 118^. Evidence of their pathogenicity stems from seminal studies in the 1950s and 1960s showing that transfer of plasma from ITP patients induced thrombocytopenia in human recipients without the disease ^112, 119^.

Following the first prospective study of rituximab in ITP in 2001 ^120^, subsequent studies have indicated a complete response rate of 44% and an overall response rate of 63% (defined as platelet count >150 and >50 × 10 ^9^ cells/L, respectively) ^121^; however, only 21–23% of patients have responses lasting five years ^122, 123^. One large randomized controlled trial in steroid-refractory patients compared rituximab to placebo with or without corticosteroids and found no difference in preventing the need for splenectomy ^124^. Similarly, a smaller pilot study examined rituximab versus placebo as adjuvants to standard care and found no difference in the composite endpoint of platelet count <50 × 10 ^9^/L, significant bleeding, or need for rescue treatment ^125^. Recent efforts to improve therapeutic efficacy have included combining rituximab with other approved ITP therapies. Three randomized controlled trials show higher rates of sustained response with combined rituximab and steroids versus steroid therapy alone, suggesting an early role for rituximab in treatment ^126– 128^. A single-arm pilot study also investigated the addition of cyclosporine to this combination, which led to a response rate of 60% with remission persisting for longer than seven months ^129^. Alternative options such as adding recombinant human thrombopoietin to rituximab have also indicated potential benefit ^130^.

### Autoimmune hemolytic anemia

In autoimmune hemolytic anemia (AIHA), the binding of antibodies to different antigens on the red blood cell (RBC) surface leads to RBC agglutination and lysis. Antibodies in warm AIHA are predominantly IgG, act at body temperature, and lead to both complement-mediated and Fc-dependent RBC destruction. In contrast, cold agglutinin disease (CAD) involves mainly IgM that bind at low temperature and result in complement-mediated RBC removal ^131^. The antibodies are highly sensitive and are required for disease diagnosis. Early demonstration of their pathogenicity came from studies on human erythrocytes sensitized with patient IgG or IgM and reintroduced into normal volunteers ^132, 133^. The role of plasma exchange in warm AIHA has not yet been established ^134^. In CAD, plasma exchange is considered a reasonable second-line option and is used in situations of elevated risk, including perioperatively and in cases of severe hemolysis ^134– 136^.

The literature in AIHA has, until recently, been limited to case reports and small prospective studies *,* with significant heterogeneity. A recent meta-analysis found an overall response rate of 73% and a complete response rate of 37%, though definitions of response differed by study ^137^. The overall and complete response rates were notably higher among warm AIHA patients (79% and 42%, respectively) compared to those with CAD (57% and 21%, respectively). Similar to studies in ITP, one open-label randomized controlled trial in warm AIHA showed that the combination of rituximab and prednisone led to a higher complete response rate at 12 months (75%) versus prednisone alone (36%), as well as a higher rate of relapse-free survival at 36 months ^138^.

### Anti-glomerular basement membrane disease

In anti-glomerular basement membrane (GBM) disease, antibodies to the alpha 3 chain of collagen IV – α3(IV) – cause rapidly progressing glomerulonephritis and pulmonary hemorrhage (Goodpasture’s syndrome), glomerulonephritis alone, or, more rarely, isolated pulmonary hemorrhage ^139, 140^. These antibodies bind rapidly and tightly to the basement membrane with slow dissociation ^141^. Newer immunoassays have shown a sensitivity of 95–100% and specificity of 90–100% ^142^. Of note, anti-α3(IV) IgG are also found in healthy individuals, although these have lower titers, reduced affinity, and different IgG subclass composition compared to those isolated from patients ^143^. Given this finding, renal biopsy in addition to antibody screening is recommended to confirm diagnosis.

The pathogenicity of anti-α3(IV) antibodies was established by the demonstration that antibodies from patient serum and from nephritic kidneys induce rapid glomerulonephritis in squirrel monkeys ^144^. Currently, standard treatment involves the expedient use of plasmapheresis combined with cyclophosphamide and corticosteroids. The addition of plasmapheresis to initial therapy has led to striking improvements in survival (from <20% to 65–100%), though response depends heavily on renal function at the initiation of therapy ^145, 146^.

Very few studies have been conducted on rituximab in anti-GBM disease. The largest case series included eight patients with severe and/or refractory disease ^147^. Seven patients achieved CR with no relapses, and rituximab was associated with a patient survival of 100%, a renal survival of 75%, and a disappearance of serum anti-GBM antibodies.

### Lambert-Eaton myasthenic syndrome

In Lambert-Eaton myasthenic syndrome (LEMS), antibodies to voltage-gated calcium channels (VGCCs) on the presynaptic terminal reduce ACh release, leading to muscle weakness and autonomic symptoms. Antibodies to the P/Q-type of VGCC are found in 85–95% of patients; they have also been observed at a lower frequency in people with malignancy, amyotrophic lateral sclerosis, and other diseases ^148– 150^. Thus, the diagnosis of LEMS requires the consideration of electrophysiologic and clinical findings along with antibody presence. Passive transfer of purified anti-VGCC antibodies into mice reproduces the electrophysiological and morphologic changes found at the neuromuscular junction in LEMS patients ^151– 153^; moreover, decreased ACh release was observed in complement-deficient mice ^154^. The autoimmune origin of LEMS has prompted the sporadic use of plasmapheresis with immunosuppression, with varying responses ^155– 158^.

Data on rituximab in LEMS come from a very small number of case reports and case series ^159, 160^. Key findings were shown in a case series that included two patients with refractory disease, both with anti-VGCC antibodies ^160^. Both patients experienced improvement but not CR.

### Epidermolysis bullosa acquisita and bullous pemphigoid

Epidermolysis bullosa acquisita (EBA) and bullous pemphigoid (BP) are subepithelial blistering diseases characterized by antibodies against different epithelial basement membrane zone antigens; specifically, EBA results from antibodies against type VII collagen (COL7), while BP arises from antibodies against BP antigen 180 (BP180, or COL17) or against BP230 ^161^. For EBA, the COL7 ELISA is highly sensitive and specific (>94%) in patients whose sera test positive with indirect immunofluorescence (IIF) to the dermal side of salt-split skin ^162– 164^. In patients with negative IIF on salt-split skin, the COL7 ELISA has high specificity (98%) but poor sensitivity (23%), leading the authors to recommend serration pattern analysis for this patient population ^165^. For BP, results of the BP180 and BP230 ELISA assays vary: most studies indicate a sensitivity and specificity of 70–98% and 90–100%, respectively, for the BP180 ELISA and 59–77% and 62–100%, respectively, for the BP230 ELISA ^166– 171^. Studies that compared the use of both the BP180 and the BP230 serologic assays to either assay alone found that the combined approach generally demonstrated improved sensitivity (87–100%) and similar specificity (88–90%) for disease diagnosis ^166, 170– 172^.

In both diseases, antibody titers correlate with disease activity ^163, 173^, and their pathogenicity is established. For EBA, rabbit IgG against murine COL7 recapitulate the EBA phenotype in mice, as do antibodies purified from patient sera. Of note, no blisters were observed with (Fab’) _2_ fragments from these same antibodies or in complement-deficient mice, indicating a critical role for Fc-mediated processes ^174, 175^. Similarly, purified human anti-BP180 antibodies and recombinant human IgG1 anti-BP180 monoclonal antibodies lead to BP-like blisters in humanized BP180 mice ^176, 177^. Fab fragments not only fail to produce this phenotype but also protect mice against autoantibody-mediated blistering ^178^. Further support for the pathogenic role of antibodies in disease comes from the use of immunoadsorption to induce remission in recalcitrant EBA and BP ^179– 182^.

Small case series in BP patients have shown favorable results with rituximab in recalcitrant disease ^183– 185^, including a study of 12 patients receiving rituximab and IVIg who all achieved both clinical and serological remission ^186^. In addition, a study of 13 BP patients found a significantly higher rate of remission with the first-line use of rituximab and corticosteroids compared to steroids alone ^187^. Larger prospective studies are needed to confirm these early results.

Data on rituximab in EBA derive mainly from case reports, making estimates of treatment response challenging. Still, rituximab has shown promising results in a few patients with refractory disease ^188– 193^, either alone or in combination with immunoadsorption ^179, 194^.

## Summary

In conclusion, the use of rituximab has led to promising results in several autoantibody-mediated diseases. Larger studies including randomized controlled trials are needed to further evaluate its effectiveness, dosing, and placement in therapeutic algorithms. Looking forward, autoantibody-mediated diseases represent an area of great potential for rituximab and other B-cell-depleting therapies.
